# Effectiveness of multi-ingredient supplement on substrate utilisation, perception of hunger, mood state and rate of perceived exertion (RPE) at rest and during exercise

**DOI:** 10.1186/1550-2783-12-S1-P42

**Published:** 2015-09-21

**Authors:** Marcos Seijo, Eneko Larumbe, Ahmad Alkhatib, Fernando Naclerio

**Affiliations:** 1 Centre for Sports Science and Human Performance, School of Science, University of Greenwich, Chatham Maritime, Kent, UK; 2 Clinical Research Institute, Texas Tech University Health Science Center, Lubbock, TX, USA; 3 Sport Science Program, College of Arts and Sciences, Qatar University, Doha, P.O. Box 2713, Qatar

## Background

Enhancing the ability to utilize fatty acids at rest and during exercise is a known important factor for weight loss and endurance performance outcomes. The aim of this study was to determine the acute effect of a multi-ingredient supplement (Shred-Matrix^®^), containing green tea extract, yerba mate, guarana seed extract, anhydrous caffeine, saw palmetto, fo-ti, eleuthero root, cayenne pepper, and yohimbine HCI, on fatty acid oxidation (FAO), perception of hunger, mood state and rate of perceived exertion (RPE) at rest and during 30 min of submaximal exercise.

## Methods

Following the ethical institutional approval and after performing an incremental test to exhaustion to determine both their peak oxygen uptake (VO_2 _peak) and the exercise intensity where fat oxidation becomes maximal (Fmax), twelve healthy recreationally active participants, 5 females and 7 males (MS ± SD age: 24 ± 3.8; Body Mass 69 ± 17.0 kg, stature 174 ± 0.09 cm) performed two experimental ergometry cycling trials 72 h apart. Following an overnight fast, participants were randomised to ingest 1.5 g (3 × capsules) of either a multi-ingredient supplement (SHRED) or placebo (PL). On both occasions, participants rested for 3 hours and then performed a constant 30-min cycling exercise test corresponding to their individually-determined Fmax intensity.

Expired gasses and stoichiometric indirect calorimetry were used to analyse fatty acid oxidation (FAO) at rest and during exercise. The rate of perceived exertion (RPE) using the Borg scale (6-20) was measured every 3 min during the 30-min exercise. Additionally both mood state and perception of hunger were assessed just after the ingestion (-3h before exercise), immediately pre and post exercise. A repeated measures ANOVA design and Cohen d effect sizes were used to analyse potential differences between times and treatment conditions.

## Results

Perception of hunger and mood state were not different between conditions. With the exception of the first 3 min time point, all RPE values were significantly lower in SHRED compared to Pl (p < 0.001). FAO increased in SHRED from -3 h to pre [0.56 (0.26) to 0.96 (0.37), p = 0.003 d 1.34] but not in PL [0.67 (0.25) to 0.74 (0.19) p = 0.334 d = 0.49]. Both conditions showed a significant increase in FAO from pre to post exercise [SHRED 0.96 (0.37) to 3.80 (1.92) p < 0.01 d = 1.72; Pl 0.74 (0.19) to 2.80 (2.02) p = 0.009 d = 1.09] with no differences between them (p = 0.12 d = 0.49).

## Conclusion

Acute ingestion of SHRED increases FAO significantly at rest, and appears to have a moderate effect size on FAO during exercise compared with PL. Those effects were combined with a significant decrease in the perception of effort during Fmax exercise intensity, but did not affect mood state and perception of hunger. The results suggest an acute effectiveness of the multi-ingredient supplement (Shred-Matrix^®^) in augmenting the weight-loss benefits at rest and during exercise.

